# Cell metabolomics analyses revealed a role of altered fatty acid oxidation in neurotoxicity pattern difference between *nab*-paclitaxel and solvent-based paclitaxel

**DOI:** 10.1371/journal.pone.0248942

**Published:** 2021-03-19

**Authors:** Jhih-Wei Huang, Ching-Hua Kuo, Han-Chun Kuo, Jin-Yuan Shih, Teng-Wen Tsai, Lin-Chau Chang

**Affiliations:** 1 School of Pharmacy, College of Medicine, National Taiwan University, Taipei City, Zhongzheng Dist., Taiwan; 2 The Metabolomics Core Laboratory, Centers of Genomic and Precision Medicine, National Taiwan University, Taipei City, Zhongzheng Dist., Taiwan; 3 Department of Internal Medicine, National Taiwan University Hospital, Taipei City, Zhongzheng Dist., Taiwan; Queen’s University Belfast, UNITED KINGDOM

## Abstract

Peripheral neuropathy (PN) is a dose-limiting, painful adverse reaction associated with the use of paclitaxel. This common side effect was often partially attributed to the solvent used for solubilization of the highly hydrophobic drug substance. Therefore, the development of alternative formulations thrived, which included that of Abraxane^®^ containing nanoparticle albumin-bound paclitaxel (*nab*-paclitaxel). However, studies demonstrated inconsistent conclusions regarding the mitigation of PN in comparison with the traditional formulation. The mass spectrometry-based cell metabolomics approach was used in the present study to explore the potentially associated mechanisms. Although no significant difference in the effects on cell viability was observed, fold changes in carnitine, several acylcarnitines and long-chain fatty acid(s) were significantly different between treatment groups in differentiated and undifferentiated SH-SY5Y cells. The most prominent difference observed was the significant increase of octanoylcarnitine in cells treated with solvent-based paclitaxel, which was found to be associated with significant decrease of medium-chain acyl-CoA dehydrogenase (MCAD). The findings suggested the potential role of altered fatty acid oxidation in the different neurotoxicity patterns observed, which may be a possible target for therapeutic interventions worth further investigation.

## Introduction

Paclitaxel is a highly effective chemotherapeutic agent with high hydrophobicity rendering the development of an injectable formulation difficult [1,2]. Many adverse reactions, including but not limited to peripheral neuropathy (PN), were partially attributed to the solvent, polyoxyl 35 castor oil, used in the traditional solvent-based formulation [1,2]. PN is a dose-limiting, painful side effect commonly observed, which may ultimately lead to treatment delays or discontinuation [2–7]. Abraxane^®^, the formulation containing nanoparticle albumin-bound paclitaxel (*nab*-paclitaxel), was then developed, which was originally expected to mitigate PN due to the elimination of the toxic solvent [1]. However, there was lack of consensus regarding its superiority in safety profiles from the aspect of PN across studies [1,8–11]. For example, no difference between these two formulations in the reporting odds ratio (ROR) for PN was found using the United States Food and Drug Administration Adverse Event Reporting System (FAERS) database [10]. Nonetheless, some studies [1,8] have demonstrated that *nab*-paclitaxel had favorable safety profiles regarding PN while others [9,11] showed conflicting results which may be due to the higher dose and dosing frequency for *nab*-paclitaxel [9].

Despite the huge impact of PN on treatment effectiveness and quality of life for patients, there is no consensus regarding the highly effective treatment or prevention strategy [3–7,12]. Although remarkable efforts were made on the understanding of the underlying mechanisms indicating the link between mitochondrial dysfunction, nitro-oxidative stress, neuroinflammation, and PN, more research is warranted in order to develop an effective approach [3–7,13,14]. Cell metabolomics involves the metabolic analysis of cell cultures, which enables the comprehensive understanding of the intermediate and end products of cellular metabolic processes in a defined cell condition with less ethics consideration than that in animal and human studies [15,16]. It facilitates more holistic analysis of metabolic responses to drug treatments and aids in the hypothesis generation and further confirmation [15,16].

Therefore, the mass spectrometry-based cell metabolomics approach was utilized in the present study and the human neuroblastoma cells, the SH-SY5Y cells, commonly used in neurotoxicity studies [17,18] were used as the model cells. To the best of our knowledge, the present study was the first report applying the cell metabolomics approach on the comparative investigation of the effects related to neurotoxicity exerted by *nab*-paclitaxel and solvent-based paclitaxel. The aim of the present study was to evaluate the metabolic profiles of the SH-SY5Y cells under the treatments of *nab*-paclitaxel and solvent-based paclitaxel so as to explore the potential underlying metabolic pathways and to search for the possible rationale for the clinically observed patterns, which may contribute to the development of strategies to alleviate paclitaxel-induced PN.

## Materials and methods

### Chemicals

Abraxane^®^ (Abraxane^®^ 5 mg/ml For Injectable Suspension) was obtained from Celgene Corporation (Summit, NJ, USA). The solvent-based formulation, Phyxol (PHYXOL Injection 6mg/ml “Sinphar”), a generic version of Taxol^®^ (paclitaxel) Injection, was purchased from Sinphar Pharmaceutical Co., Ltd. (I-Lan, Taiwan). Paclitaxel, dimethyl sulfoxide (DMSO), sulforhodamine B (SRB), L-carnitine inner salt, acetyl-DL-carnitine hydrochloride, L-carnitine-methyl-d_3_ hydrochloride, acetyl-d_3_-L-carnitine hydrochloride, phenylmethylsulfonyl fluoride (PMSF), cocktail protease inhibitor, Triton X-100, sodium bicarbonate, sodium deoxycholate, glycerol, and bromophenol blue were bought from Sigma-Aldrich (St. Louis, MO, USA). The penicillin G sodium salt/streptomycin sulfate/amphotericin B cocktail was bought from Biological Industries (Cromwell, CT, USA). Trypsin-ethylenediaminetetraacetic acid (EDTA) was obtained from Gibco (Waltham, MA, USA). Trichloroacetic acid (TCA) and mass-grade chloroform were purchased from J.T.Baker (Phillipsburg, NJ, USA). Acetic acid, glacial was obtained from Fisher Chemicals (Hampton, NH, USA). Tris(hydroxymethyl)aminomethane (Tris) was bought from Bionovas (Ontario, Canada). Mass-grade methanol and mass-grade water were purchased from Scharlau (Barcelona, Spain). Hexakis(2,2,3,3-tetrafluoropropoxy)phosphazene was purchased from Apollo Scientific (Cheshire, UK). Sodium dodecyl sulfate (SDS) and glycine were purchased from Serva (Heidelberg, Germany). Bovine serum albumin (BSA), protein assay dye reagent, β-mercaptoethanol, 30% acrylamide/bis solution (29:1), ammonium persulfate, and N,N,N’,N’-tetramethylenediamine (TEMED) were purchased from Bio-Rad (Hercules, CA, USA). High performance liquid chromatography (HPLC)-grade methanol was obtained from Honeywell (Charlotte, NC, USA). Tween 20 was purchased from Riedel-de Haën (Charlotte, NC, USA).

### The cell line and culture conditions

The human neuroblastoma cells, SH-SY5Y cells, were kindly provided by Prof. Ming-Fu Chang. SH-SY5Y cells were maintained in the Dulbecco’s modified Eagle’s medium‎ (DMEM) (Gibco, Waltham, MA, USA) supplemented with 10% fetal bovine serum (Gibco, Waltham, MA, USA), 100 units/mL penicillin G sodium salt, 100 μg/mL streptomycin sulfate, 250 ng/mL amphotericin B, 3.7 g/L sodium bicarbonate at 37°C in a humidified incubator with 5% CO_2_ in air. For experiments conducted with differentiated SH-SY5Y cells, SH-SY5Y cells were maintained in the DMEM supplemented with 10 μM of all-trans retinoic acid (RA) (Sigma-Aldrich, St. Louis, MO, USA), 5% fetal bovine serum, 100 units/mL penicillin G sodium salt, 100 μg/mL streptomycin sulfate, 250 ng/mL amphotericin B, 3.7 g/L sodium bicarbonate at 37°C in a humidified incubator with 5% CO_2_ in air for 4 days before the experiments.

### Cell viability assay

For evaluation of the cell viability of SH-SY5Y cells, the sulforhodamine B (SRB) assay [19,20] was conducted. Briefly, cells were seeded in 96-well plates at a seeding density of 4000 cells/well. Cells fixed with 10% of TCA after 24 hours represented the cell population at time zero while other cells were treated with 100 nM of paclitaxel (dissolved in DMSO), Phyxol, or Abraxane^®^ for another 24 hours before being fixed with 10% of TCA. Then, 0.4% (w/v) of SRB in 1% acetic acid was added before the addition of 1% acetic acid to wash out the unbound SRB later. The protein-bound dye was then solubilized by the addition of 10 mM of Tris. Control groups (culture media containing 0.1% DMSO, for paclitaxel group; culture media for other groups) were processed in the same manner. The absorbance (A) of the time zero group (T_0_), treatment groups (T), and control groups (C) at 515 nm would be measured using a microplate reader (SpectraMax Paradigm, Molecular Devices, San Jose, CA, USA). The cell viability would be calculated as (A_T_-A_T0_)/(A_C_-A_T0_)*100 (%). For each experiment, three samples were prepared for each condition. Three independent experiments were conducted.

### Mass spectrometry analyses of metabolic profiles

The procedure for mass spectrometry analyses of metabolic profiles was based on that previously described by Chen et al. [21] with necessary adjustments. For sample preparation, SH-SY5Y cells were seeded in 100-mm dishes at a seeding density of 10^6^ cells/dish. After 24 hours, SH-SY5Y cells were treated with 100 nM of paclitaxel (in DMSO), Phyxol, or Abraxane^®^ for 24 hours before being washed with double-distilled water (37°C) and cold mass-grade methanol (-20°C) sequentially, and collected by using cell scrapers. The solvent was removed by drying under nitrogen. A volume of 500 μL of ice-cold mass-grade methanol (4°C) was added to cell pellets, mixed at 1000 rpm for 2 minutes by using 2010 Geno/Grinder (SPEX, Metuchen, NJ, USA), and put on ice for 5 min. Then, ice-cold mass-grade water (4°C) was used instead of the methanol and the procedure was repeated. The supernatants collected by centrifugation (Centrifuge 5810R, Eppendorf, Hamburg, Germany) at 15000 g for 5 min at 4°C were filtered through a 0.2 μm regenerated cellulose membrane (Sartorius, Göttingen, Germany).

In order to conduct concentration normalization, an Agilent 1290 UHPLC system (ultra-high performance liquid chromatography) (Agilent Technologies, Santa Clara, CA, USA) coupled to an Agilent 6460 QqQ (triple quadrupole) mass spectrometry (Agilent Technologies, Santa Clara, CA, USA) was utilized. The procedure was similar to that previously described by Chen et al. [21] with necessary adjustments. The details are described in supporting information.

For analyses of the profiles of metabolites, an Agilent 1290 UHPLC system coupled to an Agilent 6540 QTOF (quadrupole time-of-flight) mass spectrometry (Agilent Technologies, Santa Clara, CA, USA) was used. A cell extract sample of 5 μL was injected into an Acquity HSS T3 column (2.1 × 100 mm, 1.8 μm, Waters, Milford, MA, USA) maintained at 40°C. The mobile phase consisted of solvent A, water/0.1% formic acid, and solvent B, acetonitrile/0.1% formic acid. The gradient elution program was as follows: 0–1.5 min, 2% B; 1.5–9 min, linear gradient from 2% to 50% B; 9–14 min, linear gradient from 50% to 95% B; hold at 95% B for 3 min. The flow rate was 300 μL min^-1^. A Jet Stream electrospray ionization source was applied for sample ionization. The mass spectrometer parameters were as follows: 325°C gas temperature, 8 L min^-1^ gas flow, 40 psi nebulizer pressure, 325°C sheath gas temperature, 10 L min^-1^ sheath gas flow, 4000 V in the positive ion mode and 3500 V in the negative ion mode for capillary voltage, and 120 V fragmentation voltage. The mass scan range was m/z 50–1700. For each experiment, three independent samples were prepared for each condition. Each sample was analyzed twice. Three independent experiments were conducted.

To further verify the signals including carnitine and acylcarnitines, an Agilent 1290 UHPLC system coupled to an Agilent 6460 QqQ mass spectrometry was used. For carnitine and acetylcarnitine, a series of L-carnitine inner salt and acetyl-DL-carnitine hydrochloride standard solutions with different known concentrations were used to establish calibration curves. Stable-isotope-labeled (SIL) analogs, L-carnitine-methyl-d_3_ hydrochloride (2000 ppb) and acetyl-d_3_-L-carnitine hydrochloride (1000 ppb), were used as internal standards. A cell extract sample of 5 μL was injected into an Acquity HSS T3 column (2.1 × 100 mm, 1.8 μm, Waters, Milford, MA, USA) maintained at 40°C. The mobile phase, the gradient elution program, and the flow rate were the same as those for aforementioned analyses of the profiles of metabolites. For sample ionization, a Jet Stream electrospray ionization source was applied. The mass spectrometer parameters were as follows: 325°C gas temperature, 7 L min^-1^ gas flow, 45 psi nebulizer pressure, 325°C sheath gas temperature, 11 L min^-1^ sheath gas flow, 3500 V capillary voltage in the positive ion mode, and 120 V fragmentation voltage. For each experiment, three independent samples were prepared for each condition. Each sample was analyzed twice. Three independent experiments were conducted.

MassHunter WorkStation Software Profinder version B.06.00 (Agilent Technologies, Santa Clara, CA, USA) was used for analyzing the profiles of metabolites obtained by using UHPLC-QTOF mass spectrometry. The in-house library with accurate masses and retention times was utilized for the identification of metabolites. The data were converted into comma-separated values (csv) format and further processed with Microsoft Excel 2016. In order to collect more reliable and reproducible signals for further analysis, the relative standard deviations of peak height values of the signals extracted were less than 25%. Meanwhile, the intensity values of the signals should be higher than blank (solvent, 50% methanol) with a difference of at least 2000. MassHunter WorkStation Software Quantitative Analysis version B.07.00 (Agilent Technologies, Santa Clara, CA, USA) was utilized for the further analysis of carnitine and acylcarnitines using UHPLC-QqQ mass spectrometry with similar data analysis procedures except that peak areas of the signals were analyzed instead of peak heights. The relative standard deviations of peak area values of the signals extracted were less than 25%. The analysis of related metabolic pathways was performed using the online tool, the Human Metabolome Database [22].

### Mass spectrometry analyses of long-chain and very long-chain fatty acids

Procedures for the collection of cell samples were similar to that mentioned before except that SH-SY5Y cells were seeded in 60-mm dishes at a seeding density of 5 × 10^5^ cells/dish. The extraction procedure and the precursor ion scan of 184 method described by Chao et al. [23] for concentration normalization were applied with necessary adjustments. Briefly, a volume of 500 μL of premixed ice-cold mass-grade methanol and mass-grade water (4°C, 4:1) was added to cell pellets, mixed at 1000 rpm for 2 minutes by using 2010 Geno/Grinder (SPEX, Metuchen, NJ, USA). Then, a volume of 200 μL of chloroform was used and the procedure was repeated before mixing with another 200 μL of chloroform by a vortex mixer (Vortex-Genie 2, Scientific Industries, Inc., Bohemia, NY, USA). The supernatants were collected by centrifugation (Centrifuge 5810R, Eppendorf, Hamburg, Germany) at 15000 g for 5 min at 4°C and then filtered through a 0.2 μm regenerated cellulose membrane (Sartorius, Göttingen, Germany). The removal of solvent was achieved by drying under nitrogen. A volume of 200 μL of mass-grade methanol was added for sample reconstitution before analysis. For concentration normalization, an Agilent 1290 UHPLC system coupled to an Agilent 6460 QqQ mass spectrometry was utilized. The details are described in supporting information.

An Agilent 1290 UHPLC system coupled to a Bruker maXis QTOF mass spectrometry (Bruker Daltonics, Bremen, Germany) was then utilized for the analyses of the profiles of long-chain and very long-chain fatty acids. A cell extract sample of 5 μL was injected into a Zorbax Eclipse Plus C18 column (2.1 × 100 mm, 1.8 μm, Agilent Technologies, Santa Clara, CA, USA) maintained at 55°C. The mobile phase contained solvent A, a mixture of 40% acetonitrile, 0.1% formic acid, 10 mM ammonium acetate, and solvent B, a mixture of isopropanol:acetonitrile (9:1), 0.1% formic acid, 10 mM ammonium acetate. The gradient elution program was as follows: 0–1 min, 10% B; 1–1.5 min, linear gradient from 10% to 40% B; 1.5–6 min, linear gradient from 40% to 70% B; 6–16 min, linear gradient from 70% to 90% B; hold at 90% B for 3 min. The flow rate was 350 μL min^-1^. An electrospray ionization source was applied for sample ionization. The mass spectrometer parameters were as follows: 200°C gas temperature, 4 L min^-1^ gas flow, 60 psi nebulizer pressure, 300°C vaporizer temperature, 2500 V capillary voltage in the positive ion mode, and 500 V end plate offset voltage. The mass scan range was m/z 50–1500. For each experiment, three independent samples were prepared for each condition. Each sample was analyzed twice. Three independent experiments were conducted.

The profile analysis was performed by Bruker Data Analysis software version 4.1 (Bruker Daltonics, Bremen, Germany). The metabolites were identified using the in-house library with accurate masses and retention times. The data were exported to.XY files and further processed with Microsoft Excel 2016. In order to collect more reliable and reproducible signals, the relative standard deviations of peak area values of the signals extracted were less than 25%.

### Western blotting

Briefly, SH-SY5Y cells were seeded in 60-mm dishes at a seeding density of 5 × 10^5^ cells/dish. After 24 hours, SH-SY5Y cells were treated with 100 nM of paclitaxel (in DMSO), Phyxol, or Abraxane^®^ for 24 hours before being harvested with trypsinization, centrifuged and lysed in the lysis buffer containing 1 mM of PMSF, 1% of cocktail protease inhibitor, 50 mM of Tris, 150 mM of sodium chloride, 1% of Triton X-100, 0.1% of SDS, and 0.5% of sodium deoxycholate. The cell lysate mixture was put on ice for 15 minutes and centrifuged at 10000 g at 4°C for 15 minutes (Centrifuge 5810R, Eppendorf, Hamburg, Germany). The supernatant was then transferred to Eppendorf tubes (Eppendorf, Hamburg, Germany). Total protein concentrations were determined using Bio-Rad protein assay kit (Bio-Rad, Hercules, CA, USA) based on Bradford protein assay principles.

The samples mixed with sample buffer (0.3 M of Tris, pH 6.8, 10% of SDS, 50% of glycerol, 10% of β-mercaptoethanol, 0.02% of bromophenol blue) were boiled at 90°C for 5 minutes. Equivalent amounts (30 μg/well) of proteins were separated with sodium dodecyl sulfate-polyacrylamide gel electrophoresis (SDS-PAGE) (Bio-Rad, Hercules, CA, USA) at 60 V, transferred to polyvinylidene difluoride (PVDF) membrane (GE Healthcare, Chicago, IL, USA) and then detected with specific antibodies. Primary antibodies included the anti-medium-chain acyl-CoA dehydrogenase (MCAD) antibody (Abcam, Cambridge, UK) and anti-cyclophilin A antibody (as a loading control) (Abcam, Cambridge, UK). Horseradish peroxidase (HRP)-conjugated anti-rabbit IgG antibody was used as the secondary antibody (Cell Signaling, Danvers, MA, USA). Protein-antibody complexes were detected and analyzed using an enhanced electrochemiluminescence reagent (PerkinElmer, Waltham, MA, USA) and ChemiDoc^TM^ MP Imaging System (Bio-Rad, Hercules, CA, USA or equivalent). The control groups (SH-SY5Y cells treated with the culture medium) were processed in the same manner.

### Statistical analysis

The one-way analysis of variance (one-way ANOVA) was performed to assess whether there were statistically significant differences among groups, followed by all pairs, Tukey HSD (honestly significant difference) post-hoc test comparisons. The statistical analysis was performed with the JMP^®^ version 14.0.0 software (SAS Institute Inc., Cary, NC, USA) and Microsoft Excel 2016 with Real Statistics Resource Pack (Release 6.7). Data are presented as mean ± standard deviation (SD). The *p* values of less than 0.05 were considered statistically significant.

## Results

### No significant difference in cell viability between cells treated with paclitaxel, Phyxol, or Abraxane^®^

**Fig 1:**
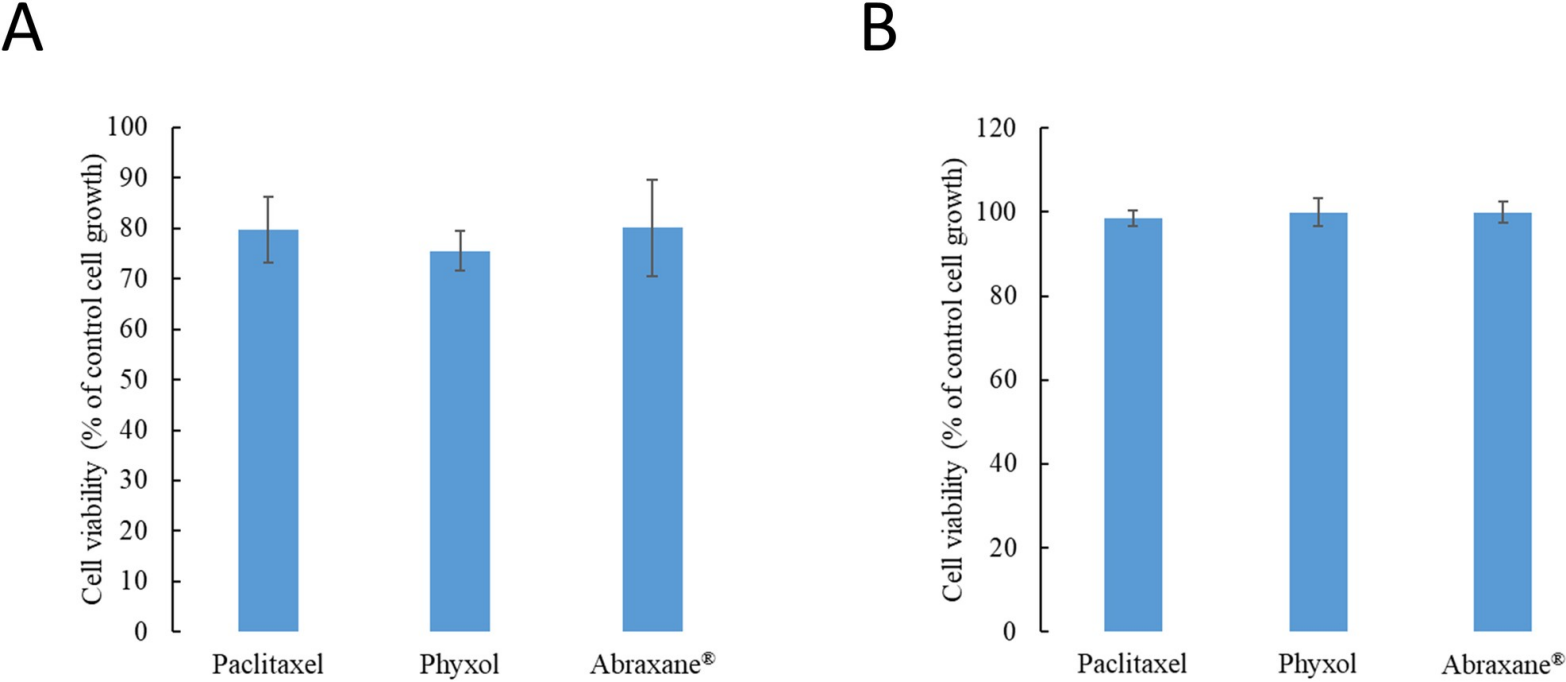
Cell viability of undifferentiated (A) and differentiated (B) SH-SY5Y cells treated with 100 nM of paclitaxel, Phyxol, or Abraxane^®^ for 24 hours assessed by the sulforhodamine B (SRB) assay. Data are expressed as mean ± standard deviation of three independent experiments.

Considering the range of serum concentrations of paclitaxel in clinical use [24], the concentration, 100 nM, was used in the present studies evaluating the safety impact at this effective concentration. No significant difference in cell viability was found between SH-SY5Y cells treated with paclitaxel, Phyxol, or Abraxane^®^ after 24 hours regardless of the differentiation status (Fig 1). For differentiated SH-SY5Y cells, the cell viability was similar to that of the control groups (Fig 1B), suggesting that the cell viability of the differentiated SH-SY5Y cells may not be largely affected by the tubulin stabilization effect of paclitaxel under the test condition.

### Altered level changes of carnitine and certain acylcarnitines between treatment groups

The UHPLC-QTOF mass spectrometry was applied to search for potential difference between the metabolic profiles of SH-SY5Y cells treated with 100 nM of paclitaxel (in DMSO), Phyxol, or Abraxane^®^ for 24 hours in comparison with those of the control group. Among 43 and 67 detected metabolites in undifferentiated and differentiated SH-SY5Y cells (S1 and S2 Tables), only 7 and 6 metabolites, respectively, showed significant difference in the fold changes normalized to the control group between treatment groups indicating the general similarity of effects exerted by these formulations (Tables 1 and 2). Importantly, carnitine (*p* = 0.0318) and several acylcarnitines (acetylcarnitine, *p* = 0.0367; propionylcarnitine, *p* = 0.0328; butyrylcarnitine, *p* = 0.0013; valerylcarnitine, *p* = 0.0139) showed significant difference in fold changes in undifferentiated SH-SY5Y cells (Table 1). Fold changes in carnitine were also significantly different (*p* = 0.0123) between treatment groups in differentiated SH-SY5Y cells (Table 2).

**Table 1:** Metabolic profiles of undifferentiated SH-SY5Y cells treated with 100 nM of paclitaxel (in dimethyl sulfoxide), Phyxol, or Abraxane^®^^a^.

Retention time (min)	Metabolite^b^	Fold change (n = 3)^c^			*p* value^d^			
		PTX/CTL (A)	PHY/CTL (B)	ABR/CTL (C)	Total	A versus B	A versus C	B versus C
0.77	Arginine	1.24 ± 0.08	0.78 ± 0.01	0.62 ± 0.08	<0.0001***	0.0003***	<0.0001***	0.0344*
0.83	Carnitine	0.68 ± 0.12	0.76 ± 0.09	0.94 ± 0.06	0.0318*	0.5246	0.0289*	0.1189
0.90	Glycerol 3-phosphate	0.77 ± 0.03	0.78 ± 0.11	0.96 ± 0.05	0.0270*	0.0367*	0.9839	0.0452*
1.36	Acetylcarnitine	0.71 ± 0.09	0.68 ± 0.11	0.90 ± 0.02	0.0367*	0.9224	0.0703	0.0436*
2.67	Propionylcarnitine	0.76 ± 0.10	0.82 ± 0.08	1.04 ± 0.12	0.0328*	0.7678	0.0341*	0.0817
4.37	Butyrylcarnitine	0.75 ± 0.05	0.58 ± 0.04	0.89 ± 0.07	0.0013**	0.0200*	0.0438*	0.0010**
5.34	Valerylcarnitine	0.66 ± 0.05	0.72 ± 0.09	0.87 ± 0.02	0.0139*	0.5127	0.0132*	0.0514

^a^ ABR, Abraxane^®^; CTL, control; PHY, Phyxol; PTX, paclitaxel; RT, retention time

*, *p* < 0.05

**, *p* < 0.01

***, *p* < 0.001.

^b^ Since isomers could not be distinguished by the present analytical method, the metabolites listed herein were representative. Only metabolites with significant difference in the fold changes normalized to the control group between treatment groups are displayed.

^c^ Three independent samples were prepared for each condition in each experiment. Each sample was analyzed twice by ultra-high performance liquid chromatography-quadrupole time-of-flight mass spectrometry. Three independent experiments were conducted. Data are expressed as the fold change relative to the control group and are presented as mean ± standard deviation (n = 3).

^d^ One-way analysis of variance was used to analyze the difference in fold changes; all pairs, Tukey HSD (honestly significant difference) post-hoc test comparisons were then performed.

**Table 2:** Metabolic profiles of differentiated SH-SY5Y cells treated with 100 nM of paclitaxel (in dimethyl sulfoxide), Phyxol, or Abraxane^®^^a^.

Retention time (min)	Metabolite^b^	Fold change (n = 3)^c^			*p* value^d^			
		PTX/CTL (A)	PHY/CTL (B)	ABR/CTL (C)	Total	A versus B	A versus C	B versus C
0.83	Carnitine	0.84 ± 0.05	0.96 ± 0.07	1.02 ± 0.02	0.0123*	0.0659	0.0107*	0.3281
1.96	Tyrosine	0.94 ± 0.03	1.06 ± 0.05	1.11 ± 0.07	0.0195*	0.0645	0.0188*	0.5847
4.73	Tryptophan	0.95 ± 0.02	1.00 ± 0.02	1.02 ± 0.03	0.0294*	0.1058	0.0270*	0.5429
4.74	1-Phenylethylamine	0.91 ± 0.04	1.00 ± 0.01	1.11 ± 0.10	0.0190*	0.2401	0.0158*	0.1448
4.74	3-Methylglutaric acid	0.91 ± 0.03	0.98 ± 0.05	1.11 ± 0.07	0.0105*	0.3559	0.0094**	0.0518
4.84	γ-Glutamylleucine	0.93 ± 0.07	1.08 ± 0.04	1.07 ± 0.06	0.0360*	0.0447*	0.0645	0.9530

^a^ ABR, Abraxane^®^; CTL, control; PHY, Phyxol; PTX, paclitaxel; RT, retention time

*, *p* < 0.05

**, *p* < 0.01.

^b^ Since isomers could not be distinguished by the present analytical method, the metabolites listed herein were representative. Only metabolites with significant difference in the fold changes normalized to the control group between treatment groups are displayed.

^c^ Three independent samples were prepared for each condition in each experiment. Each sample was analyzed twice by ultra-high performance liquid chromatography-quadrupole time-of-flight mass spectrometry. Three independent experiments were conducted. Data are expressed as the fold change relative to the control group and are presented as mean ± standard deviation (n = 3).

^d^ One-way analysis of variance was used to analyze the difference in fold changes; all pairs, Tukey HSD (honestly significant difference) post-hoc test comparisons were then performed.

**Fig 2:**
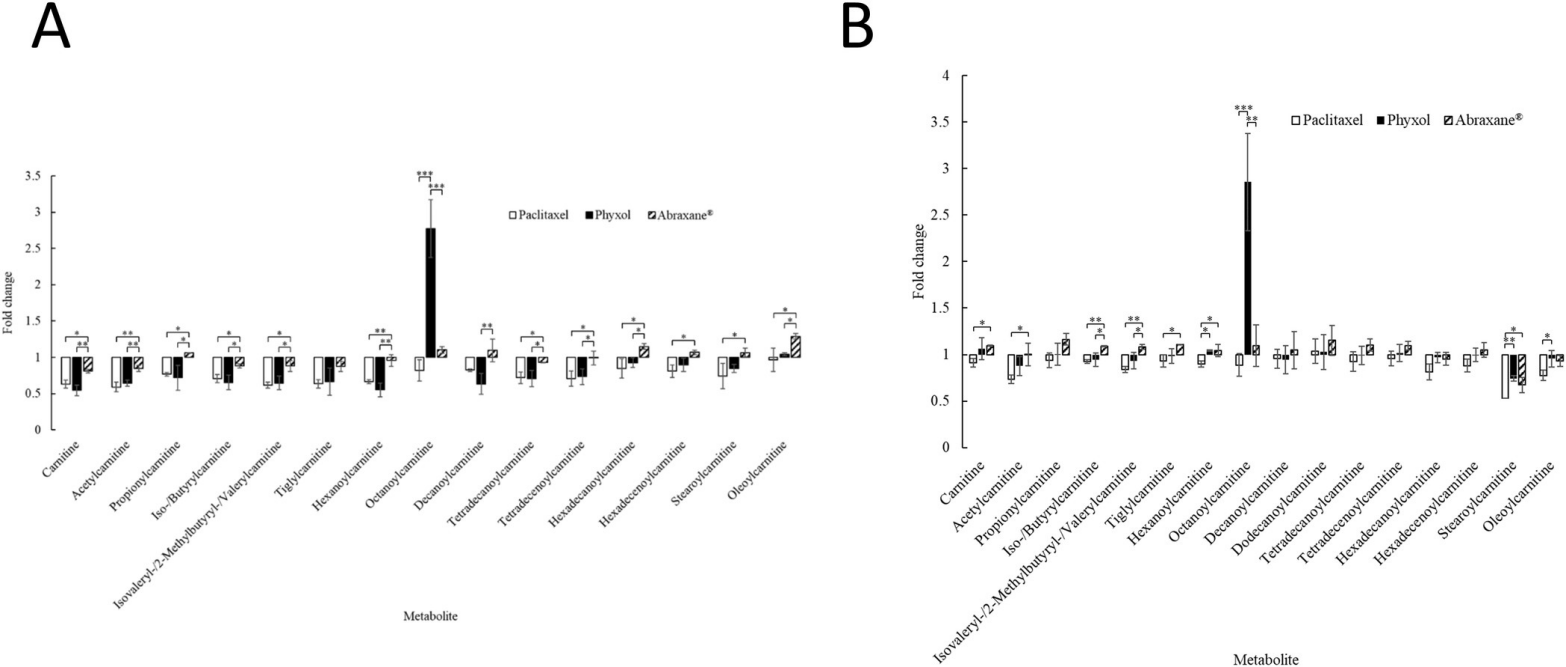
Fold changes relative to the control group in the levels of carnitine and acylcarnitines of undifferentiated (A) and differentiated (B) SH-SY5Y cells treated with 100 nM of paclitaxel (in dimethyl sulfoxide), Phyxol, or Abraxane^®^ for 24 hours. Three independent samples were prepared for each condition in each experiment. Each sample was analyzed twice by ultra-high performance liquid chromatography-triple quadrupole mass spectrometry. Three independent experiments were conducted. Calibration curves of the standards and the internal standards for carnitine and acetylcarnitine were used for quantitation of these two metabolites. Data are presented as mean ± standard deviation. One-way analysis of variance was used to analyze the difference in fold changes between treatment groups for each metabolite. If the difference was significant, all pairs, Tukey HSD (honestly significant difference) post-hoc test comparisons were performed. *, *p* < 0.05; **, *p* < 0.01; ***, *p* < 0.001.

For further verification of the signals for carnitines and acylcarnitines, UHPLC-QqQ mass spectrometry was utilized. Among these metabolites, the quantitation of carnitine and acetylcarnitine was performed by using calibration curves of the standards and the internal standards. In undifferentiated SH-SY5Y cells, levels of carnitine and short-chain acylcarnitines (the number of carbon atoms for the acyl group: 2–5) [25] were decreased in the treatment groups except for propionylcarnitine in the cells treated with Abraxane^®^ (Fig 2A). The extent of the decrease was smallest in the cells treated with Abraxane^®^ (Fig 2A). Significant difference in the fold changes relative to the control group could be found between cells treated with Phyxol and those treated with Abraxane^®^ except for tiglylcarnitine (Fig 2A).

For medium-chain acylcarnitines (the number of carbon atoms for the acyl group: 6–12) [25], the prominent increase in the level of octanoylcarnitine in cells treated with Phyxol (Fig 2A) was worth noticing as that was often associated with the deficiency of medium-chain acyl-CoA dehydrogenase (MCAD) [26]. Significant difference in the fold changes relative to the control group could also be found between cells treated with Phyxol and those treated with Abraxane^®^ (Fig 2A). For long-chain acylcarnitines (the number of carbon atoms for the acyl group: 14–18) [25], the dissimilar behavior between cells treated with Phyxol and those treated with Abraxane^®^ was also obvious (Fig 2A).

In differentiated SH-SY5Y cells, the trend for level changes in carnitine and acylcarnitines was not totally the same with that observed in undifferentiated SH-SY5Y cells (Fig 2). Between cells treated with Phyxol and those treated with Abraxane^®^, significant difference in the fold changes relative to the control group could only be found in iso-/butyrylcarnitine, isovaleryl-/2-methylbutyryl-/valerylcarnitine, and octanoylcarnitine (Fig 2B). Nonetheless, there was also a noteworthy increase in the level of octanoylcarnitine in cells treated with Phyxol (Fig 2B). Results regarding the metabolic profiles have revealed that the formulation change could have different impact on SH-SY5Y cells and the influence depended on the differentiation status.

### More obvious difference of the impact on level changes of long-chain fatty acids between treatment groups in undifferentiated SH-SY5Y cells

**Fig 3:**
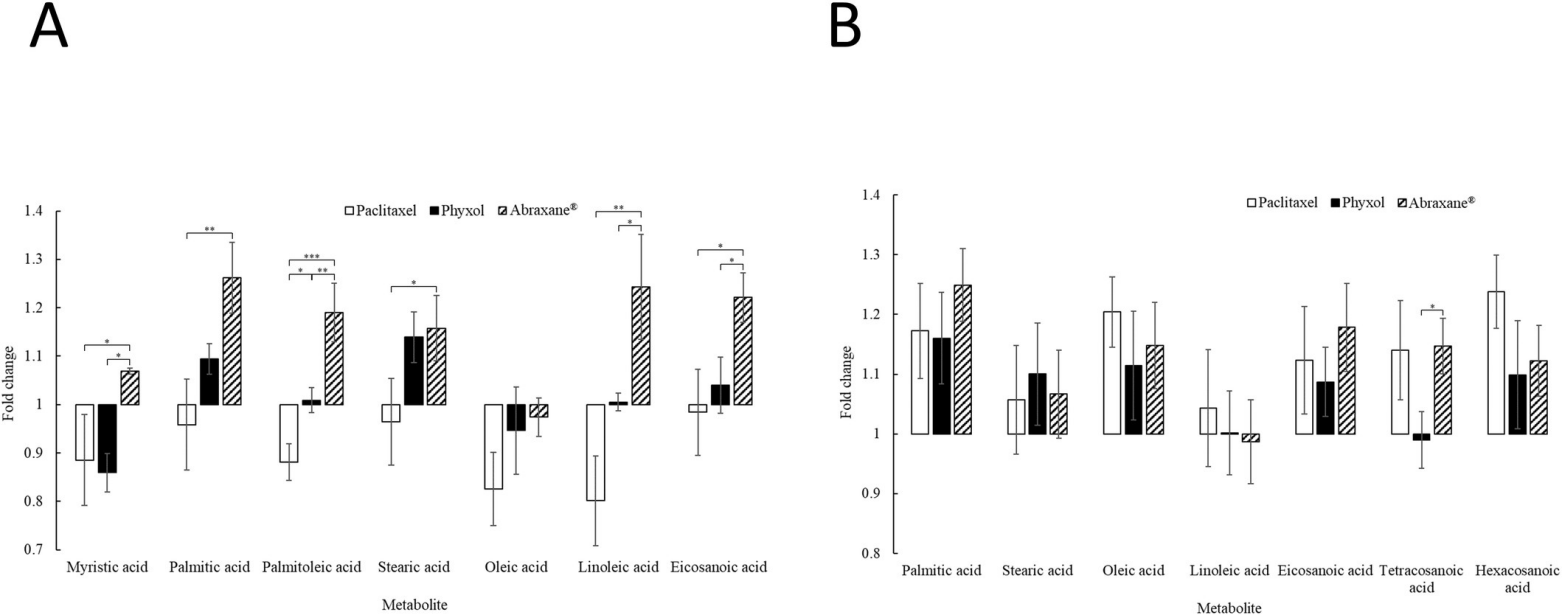
Fold changes relative to the control group in the levels of long-chain and very long-chain fatty acids of undifferentiated (A) and differentiated (B) SH-SY5Y cells treated with 100 nM of paclitaxel (in dimethyl sulfoxide), Phyxol, or Abraxane^®^ for 24 hours. Three independent samples were prepared for each condition in each experiment. Each sample was analyzed twice by ultra-high performance liquid chromatography-quadrupole time-of-flight mass spectrometry. Three independent experiments were conducted. Data are presented as mean ± standard deviation. One-way analysis of variance was used to analyze the difference in fold changes between treatment groups for each metabolite. If the difference was significant, all pairs, Tukey HSD (honestly significant difference) post-hoc test comparisons were performed. *, *p* < 0.05; **, *p* < 0.01; ***, *p* < 0.001.

Since long-chain fatty acids require carnitine system for the transportation into mitochondria for beta oxidation [27], the levels of long-chain fatty acids in undifferentiated SH-SY5Y cells treated with 100 nM of paclitaxel (in DMSO), Phyxol, or Abraxane^®^ for 24 hours were further assessed considering the difference in the level changes of carnitine and several long-chain acylcarnitines between treatment groups. Those in differentiated SH-SY5Y cells were also evaluated for comparison. In undifferentiated SH-SY5Y cells, the levels of long-chain acylcarnitines (Fig 2A) and long-chain fatty acids (Fig 3A) observed in cells treated with Abraxane^®^ were higher than those in cells treated with Phyxol. For long-chain fatty acids, significant difference in the fold changes relative to the control group between cells treated with Phyxol and those treated with Abraxane^®^ was found in myristic acid, palmitoleic acid, linoleic acid, and eicosanoic acid (Fig 3A). Eicosanoic acid may sometimes be classified as very long-chain fatty acids since the definition for very long-chain fatty acids varied, which may include fatty acids with the carbon chain length equal to and above 20, 22, 24 or 26 [28]. In differentiated SH-SY5Y cells, the pattern (Fig 3B) was different from that for undifferentiated SH-SY5Y cells (Fig 3A). Significant difference in the fold changes relative to the control group between cells treated with Phyxol and those treated with Abraxane^®^ could only be found in tetracosanoic acid (Fig 3B) among the reproducible signals detected.

### Decreased medium-chain acyl-CoA dehydrogenase (MCAD) levels in SH-SY5Y cells treated with Phyxol

**Fig 4:**
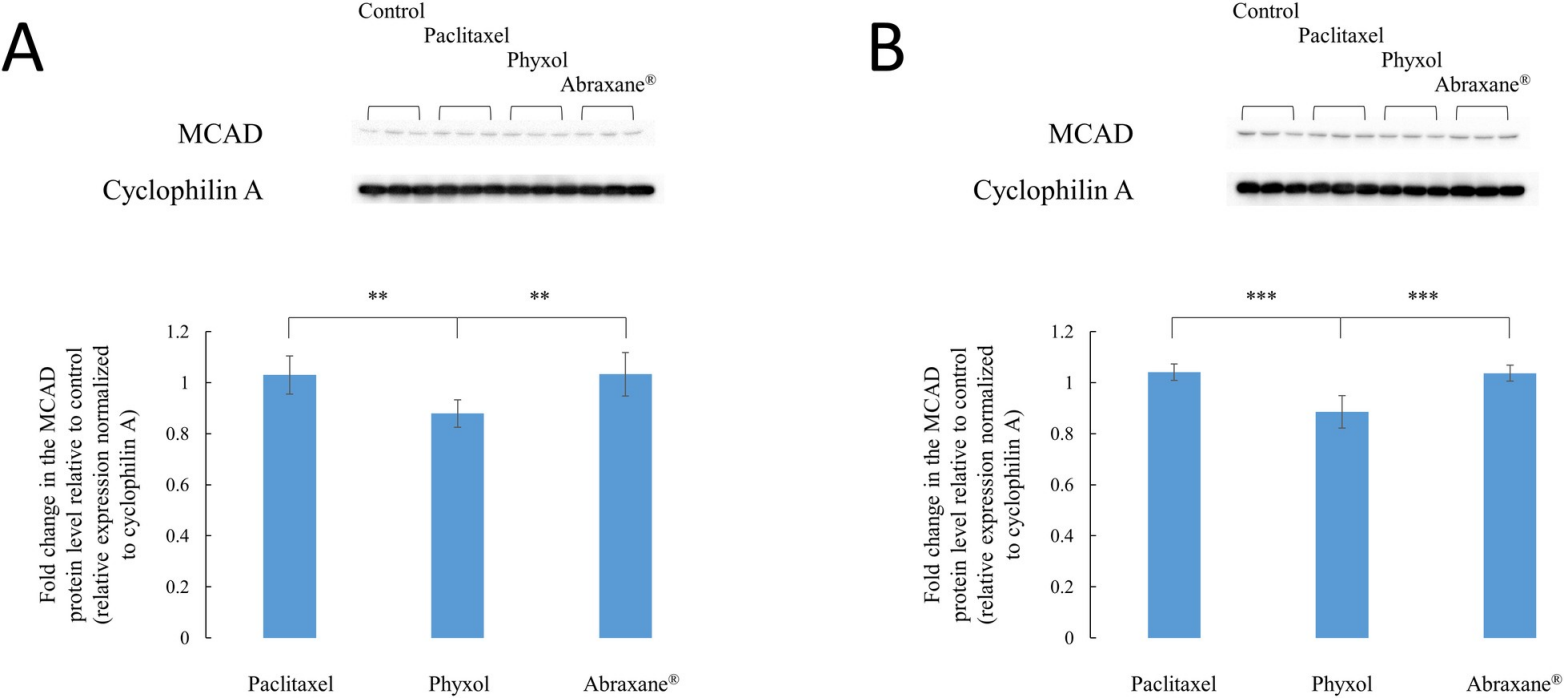
Expression of medium-chain acyl-CoA dehydrogenase (MCAD) proteins in undifferentiated (A) and differentiated (B) SH-SY5Y cells treated with 100 nM of paclitaxel (in dimethyl sulfoxide), Phyxol, or Abraxane^®^ for 24 hours. Upper panels show the representative Western blot images, while lower panels demonstrate the fold change relative to the control group, which are presented as mean ± standard deviation. Six independent samples were prepared for each condition. One-way analysis of variance was used to analyze the difference in fold changes; all pairs, Tukey HSD (honestly significant difference) post-hoc test comparisons were then performed. **, *p* < 0.01; ***, *p* < 0.001.

Considering the association between the increase in octanoylcarnitine and the deficiency of MCAD [26], protein levels of MCAD were assessed. Both undifferentiated and differentiated SH-SY5Y cells showed significantly decreased expression of MCAD under treatment of Phyxol in comparison with other treatment groups (Fig 4). This finding corresponded to the significant increase in the levels of octanoylcarnitine for cells treated with Phyxol (Fig 2).

## Discussion

Ever since the success of paclitaxel in the treatment of many advanced and refractory cancers, efforts have been made to develop alternative formulations to eliminate the use of the solvent, polyoxyl 35 castor oil, to which many adverse reactions including the exacerbation of PN have been attributed [2]. For example, Zang et al. [29] recently showed that a liposome formulation of paclitaxel resulted in significantly less sensitivity of rats to mechanical and thermal stimuli in comparison with that induced by the traditional formulation (Taxol^®^). The findings indicated the potential of reducing neurotoxicity through the application of nanoformulations [29].

Despite the elimination of the toxic solvent in the novel formulation, Abraxane^®^, studies demonstrated inconsistent conclusions regarding the mitigation of PN in comparison with traditional, solvent-based paclitaxel [1,8–11]. Through the application of cell metabolomics approach in the present study, Abraxane^®^ and Phyxol differentially modulated levels of carnitine and acylcarnitines. A recent systematic review investigating evidence from clinical trials suggested that the administration of acetyl-L-carnitine was promising in treating PN [12], while Wang et al. [30] demonstrated that L-carnitine ameliorated PN in diabetic mice. In the present study, the extent of decrease (relative to control) in the levels of carnitine and acetylcarnitine in undifferentiated SH-SY5Y cells treated with Abraxane^®^ was significantly lower than that in cells treated with paclitaxel or Phyxol (Fig 2A). In differentiated SH-SY5Y cells, results also demonstrated that the levels of carnitine and acetylcarnitine were significantly higher in cells treated with Abraxane^®^ than that in cells treated with paclitaxel, while no significant difference in the levels of carnitine and acetylcarnitine between cells treated with Phyxol and those treated with paclitaxel could be found (Fig 2B). These findings may support the previous studies showing that *nab*-paclitaxel had favorable safety profiles in comparison with the traditional formulation [1,8].

While the carnitine system and fatty acid oxidation are intimately correlated [27,31], fatty acid oxidation disorders have been related to a wide spectrum of clinical manifestations such as progressive lipid storage myopathy, recurrent myoglobinuria, and neuropathy [27,31]. In the present study, the differential impact on fatty acid oxidation exerted by different formulations of paclitaxel was obvious, leading to dissimilar metabolic profiles with varied levels of carnitine, acylcarnitines, and long-chain fatty acids as observed herein. The most prominent difference observed was the significant increase of octanoylcarnitine in cells treated with Phyxol in comparison with that in cells treated with paclitaxel or Abraxane^®^ (Fig 2), which was found to be associated with significant decrease of MCAD (Fig 4). The relationship between the increase of octanoylcarnitine and the decrease of MCAD was supported by previous studies investigating the deficiency of MCAD [26]. It is suspected that the excipients, especially polyoxyl 35 castor oil, in Phyxol played an important role in eliciting this response. Further investigation is worthy to elucidate the individual effect of each excipient.

Regarding the relationship between acyl-CoA dehydrogenase deficiency and neuropathy, Wang et al. [32] reported that severe sensory neuropathy has been presented in six patients with adult-onset multiple acyl-CoA dehydrogenase deficiency and mutations of the *ETFDH* gene. They further indicated the possible association between mitochondrial dysfunction, metabolic disturbance of acylcarnitines, and sensory neuropathy, although the precise mechanism was unclear [32]. Our findings also suggested that the neuropathy observed with the use of Phyxol might be associated with decreased MCAD expression and metabolic perturbations in carnitine and acylcarnitines, which may reinforce the statement of Wang et al [32].

In the present study, both undifferentiated and differentiated SH-SY5Y cells commonly used in neurobiology investigation [17,18,33] were used as the model cell lines for investigation. Undifferentiated SH-SY5Y cells lacking mature neuronal markers were considered to more resemble immature catecholaminergic neurons, while the addition of RA was known to cause SH-SY5Y cells to differentiate primarily to a cholinergic neuron phenotype [33]. Cheung et al. [17] showed previously that RA-induced differentiation rendered SH-SY5Y cells less susceptible to several neurotoxins in comparison with the undifferentiated ones. Herein, we also observed higher tolerance against paclitaxel in RA-induced differentiated SH-SY5Y cells (Fig 1). Although undifferentiated and differentiated SH-SY5Y cells may differ in terms of their characteristics and representativeness for serving as a model for PN, [17,18,33] results from both cells indicated the discrepant effect on fatty acid oxidation exerted by different paclitaxel formulations.

## Conclusions

In conclusion, the cell metabolomics approach was applied in the present study to compare the metabolic perturbations caused by *nab*-paclitaxel and solvent-based paclitaxel. Difference in metabolic perturbations included but were not limited to carnitine and acylcarnitines, among which the significant increases in the levels of octanoylcarnitine in both undifferentiated and differentiated SH-SY5Y cells treated with solvent-based paclitaxel were further found to be associated with the significant decrease in the expression of MCAD. Although the detailed mechanisms and the breakthrough treatment for PN require further investigation, the present findings may signify the importance of fatty acid oxidation in the difference observed from the use of *nab*-paclitaxel and solvent-based paclitaxel, which may represent a possible target for therapeutic interventions. Moreover, the present study also demonstrated the potential of using cell metabolomics approach in the investigation of the impact of formulations.

## Supporting information

S1 Table Metabolic profiles of undifferentiated SH-SY5Y cells treated with 100 nM of paclitaxel (in dimethyl sulfoxide), Phyxol, or Abraxane^®^. (PDF)

S2 Table Metabolic profiles of differentiated SH-SY5Y cells treated with 100 nM of paclitaxel (in dimethyl sulfoxide), Phyxol, or Abraxane^®^. (PDF)

S1 File Original images for Fig 4. (PDF)

S2 File Details regarding experimental procedures. Data for figures.
(PDF)
