# Supplementary material for: Cell metabolomics analyses revealed a role of altered fatty acid oxidation in neurotoxicity pattern difference between nab-paclitaxel and solvent-based paclitaxel
Source: PLoS One. 2021 Mar 19;16(3):e0248942. doi: 10.1371/journal.pone.0248942 (PMC7978375; doi:10.1371/journal.pone.0248942)
Supplement: S1 Table — (PDF) [file pone.0248942.s001.pdf]

S1 Table. Metabolic profiles of undifferentiated SH-SY5Y cells treated with 100 nM of paclitaxel (in dimethyl sulfoxide), Phyxol, or Abraxane®[a].

Retention time (min)	Metabolite[b]	Fold change (n=3)[c]			*p* value[d]			
		PTX/CTL (A)	PHY/CTL (B)	ABR/CTL (C)	Total	A versus B	A versus C	B versus C
0.69	1-Methylhistidine	0.97 ± 0.14	1.06 ± 0.12	1.04 ± 0.05	0.6333	0.6325	0.7560	0.9740
0.76	Histidine	0.91 ± 0.02	0.90 ± 0.06	1.00 ± 0.06	0.0911	0.9414	0.1572	0.1028
0.77	Arginine	1.24 ± 0.08	0.78 ± 0.01	0.62 ± 0.08	<0.0001***	0.0003***	<0.0001***	0.0344*
0.80	Asparagine	0.96 ± 0.05	0.96 ± 0.05	0.97 ± 0.10	0.9605	0.9930	0.9571	0.9843
0.81	Alanine	0.78 ± 0.02	0.73 ± 0.09	0.81 ± 0.10	0.4541	0.7161	0.8580	0.4286
0.81	Aspartic acid	0.97 ± 0.08	0.94 ± 0.11	1.04 ± 0.11	0.5119	0.8988	0.7354	0.4899

0.82	Glutamine	1.03 ± 0.09	0.96 ± 0.20	1.01 ± 0.14	0.8049	0.7980	0.9803	0.8910
0.82	Taurine	0.89 ± 0.05	0.81 ± 0.09	0.74 ± 0.20	0.4212	0.7329	0.3925	0.8032
0.83	5-Aminolevulinic acid	0.86 ± 0.12	0.84 ± 0.03	0.94 ± 0.09	0.3709	0.9095	0.5655	0.3610
0.83	Carnitine	0.68 ± 0.12	0.76 ± 0.09	0.94 ± 0.06	0.0318*	0.5246	0.0289*	0.1189
0.83	Fructose	1.23 ± 0.03	1.25 ± 0.19	1.03 ± 0.05	0.1120	0.9756	0.1733	0.1322
0.83	Glycerophosphocholine	1.16 ± 0.14	1.17 ± 0.08	1.17 ± 0.17	0.9981	0.9995	0.9979	0.9995
0.83	Threonine	0.93 ± 0.11	0.85 ± 0.09	0.94 ± 0.10	0.5102	0.6471	0.9687	0.5145
0.84	5-Methylcytidine	1.16 ± 0.14	1.17 ± 0.07	1.17 ± 0.17	0.9981	0.9995	0.9979	0.9995
0.84	Glutamic acid	0.82 ± 0.10	0.78 ± 0.08	0.87 ± 0.17	0.6584	0.9285	0.8401	0.6373
0.84	Mannitol	0.88 ± 0.10	0.88 ± 0.10	0.90 ± 0.19	0.9662	0.9995	0.9685	0.9758
0.85	Creatinine	1.00 ± 0.12	0.92 ± 0.23	0.72 ± 0.06	0.1570	0.7961	0.1489	0.3360

0.85	Orotic acid	1.18 ± 0.03	1.16 ± 0.13	0.92 ± 0.19	0.1029	0.9878	0.1268	0.1534
0.85	Ribose	0.75 ± 0.08	0.74 ± 0.06	0.83 ± 0.04	0.2461	0.9608	0.3615	0.2617
0.87	Orotidine	0.87 ± 0.13	0.77 ± 0.16	1.00 ± 0.13	0.2191	0.6742	0.5438	0.1970
0.89	Creatine	1.03 ± 0.04	1.09 ± 0.09	0.98 ± 0.05	0.1964	0.5167	0.6514	0.1756
0.89	Ribose 5-phosphate	0.86 ± 0.17	0.84 ± 0.18	1.02 ± 0.10	0.3631	0.9939	0.4508	0.4013
0.90	Glycerol 3-phosphate	0.77 ± 0.03	0.78 ± 0.11	0.96 ± 0.05	0.0270*	0.0367*	0.9839	0.0452*
0.91	Glycerone phosphate	0.80 ± 0.12	0.78 ± 0.18	0.96 ± 0.05	0.2291	0.9795	0.3219	0.2545
0.98	Valine	0.86 ± 0.13	0.98 ± 0.08	0.99 ± 0.04	0.2488	0.3227	0.2895	0.9955
1.20	Malic acid	0.70 ± 0.13	0.64 ± 0.14	0.77 ± 0.07	0.4556	0.7884	0.7884	0.4259
1.20	Pyruvic acid	0.72 ± 0.13	0.65 ± 0.13	0.77 ± 0.08	0.4953	0.7555	0.8638	0.4687
1.36	Acetylcarnitine	0.71 ± 0.09	0.68 ± 0.11	0.90 ± 0.02	0.0367*	0.9224	0.0703	0.0436*

1.38	Lactate	1.08 ± 0.16	1.08 ± 0.13	1.00 ± 0.09	0.7092	0.9977	0.7696	0.7341
1.39	Methionine	1.01 ± 0.01	0.96 ± 0.17	1.01 ± 0.19	0.8821	0.9012	1.0000	0.9012
1.84	Dopamine	0.93 ± 0.06	1.05 ± 0.16	0.98 ± 0.10	0.4864	0.4581	0.8423	0.7681
1.93	Isoleucine	1.02 ± 0.07	1.00 ± 0.14	1.00 ± 0.10	0.9699	0.9711	0.9798	0.9992
1.96	Tyrosine	0.96 ± 0.10	0.92 ± 0.14	1.04 ± 0.08	0.4173	0.8652	0.6659	0.3949
2.16	Norleucine	1.04 ± 0.04	0.99 ± 0.07	1.05 ± 0.05	0.3500	0.4785	0.9672	0.3640
2.67	Propionylcarnitine	0.76 ± 0.10	0.82 ± 0.08	1.04 ± 0.12	0.0328*	0.7678	0.0341*	0.0817
3.80	Phenylalanine	1.03 ± 0.02	0.97 ± 0.05	1.01 ± 0.07	0.5044	0.4815	0.8918	0.7353
4.37	Butyrylcarnitine	0.75 ± 0.05	0.58 ± 0.04	0.89 ± 0.07	0.0013**	0.0200*	0.0438*	0.0010**
4.73	Tryptophan	0.98 ± 0.13	0.96 ± 0.14	1.05 ± 0.09	0.5908	0.9777	0.7106	0.5959
4.85	4-Hydroxybenzaldehyde	0.89 ± 0.05	0.86 ± 0.10	1.07 ± 0.14	0.0856	0.9190	0.1558	0.0943

4.85	Homovanillate	0.89 ± 0.06	0.86 ± 0.10	1.07 ± 0.14	0.0864	0.9349	0.1521	0.0971
5.34	Valerylcarnitine	0.66 ± 0.05	0.72 ± 0.09	0.87 ± 0.02	0.0139*	0.5127	0.0132*	0.0514
5.43	Ketoleucine	1.08 ± 0.09	1.09 ± 0.12	1.11 ± 0.10	0.9711	0.9965	0.9693	0.9862
5.81	2-Ketohexanoic acid	1.04 ± 0.12	1.09 ± 0.14	1.15 ± 0.08	0.5644	0.8786	0.5377	0.8098

[a] ABR, Abraxane®; CTL, control; PHY, Phyxol; PTX, paclitaxel; RT, retention time; *, $p < 0.05$; **, $p < 0.01$; ***, $p < 0.001$.

[b] Since isomers could not be distinguished by the present analytical method, the metabolites listed herein were representative.

[c] Three independent samples were prepared for each condition in each experiment. Each sample was analyzed twice by ultra-high performance liquid chromatography-quadrupole time-of-flight mass spectrometry. Three independent experiments were conducted. Data are expressed as the fold change relative to the control group and are presented as mean ± standard deviation (n = 3).

[d] One-way analysis of variance was used to analyze the difference in fold changes; all pairs, Tukey HSD (honestly significant difference) post-hoc test comparisons were then performed.
