# Supplementary material for: Cell metabolomics analyses revealed a role of altered fatty acid oxidation in neurotoxicity pattern difference between nab-paclitaxel and solvent-based paclitaxel
Source: PLoS One. 2021 Mar 19;16(3):e0248942. doi: 10.1371/journal.pone.0248942 (PMC7978375; doi:10.1371/journal.pone.0248942)
Supplement: S2 Table — (PDF) [file pone.0248942.s002.pdf]

S2 Table. Metabolic profiles of differentiated SH-SY5Y cells treated with 100 nM of paclitaxel (in dimethyl sulfoxide), Phyxol, or Abraxane®[a].

Retention time (min)	Metabolite[b]	Fold change (n=3)[c]			*p* value[d]			
		PTX/CTL (A)	PHY/CTL (B)	ABR/CTL (C)	Total	A versus B	A versus C	B versus C
0.68	Guanidine	1.07 ± 0.06	1.13 ± 0.15	1.33 ± 0.20	0.1603	0.8573	0.1582	0.3080
0.70	Lysine	0.86 ± 0.04	0.91 ± 0.11	0.90 ± 0.03	0.5995	0.6175	0.6945	0.9899
0.70	Ornithine	0.84 ± 0.05	0.91 ± 0.09	0.87 ± 0.02	0.4543	0.4277	0.8486	0.7260
0.71	Cotinine	1.02 ± 0.11	1.07 ± 0.24	1.05 ± 0.20	0.9601	0.9569	0.9834	0.9934
0.79	Fructose	0.97 ± 0.09	1.00 ± 0.14	0.93 ± 0.19	0.8413	0.9785	0.9216	0.8321
0.79	Glycine	0.90 ± 0.08	0.85 ± 0.13	0.81 ± 0.08	0.5470	0.7825	0.5218	0.8883
0.80	Asparagine	0.89 ± 0.13	0.88 ± 0.09	0.81 ± 0.06	0.5470	0.9952	0.5788	0.6309
0.80	Serine	0.88 ± 0.06	0.97 ± 0.05	0.83 ± 0.08	0.0721	0.2133	0.6470	0.0661
0.81	Alanine	0.91 ± 0.03	0.91 ± 0.02	0.89 ± 0.01	0.5892	1.0000	0.6406	0.6436

0.81	Aspartic acid	0.95 ± 0.04	0.95 ± 0.06	0.97 ± 0.03	0.9025	0.9911	0.8990	0.9468
0.82	Glutamine	1.11 ± 0.26	0.74 ± 0.16	0.76 ± 0.13	0.1008	0.1276	0.1463	0.9937
0.82	Taurine	0.94 ± 0.06	0.90 ± 0.10	0.88 ± 0.09	0.6864	0.8328	0.6716	0.9537
0.83	5-Aminolevulinic acid	0.92 ± 0.11	0.91 ± 0.10	0.86 ± 0.05	0.6751	0.9821	0.6779	0.7792
0.83	Carnitine	0.84 ± 0.05	0.96 ± 0.07	1.02 ± 0.02	0.0123*	0.0659	0.0107*	0.3281
0.83	Glycerophosphocholine	0.99 ± 0.02	1.00 ± 0.01	1.00 ± 0.01	0.9073	0.9621	0.8997	0.9831
0.83	Threonine	0.89 ± 0.03	0.90 ± 0.10	0.88 ± 0.03	0.8940	0.9872	0.9471	0.8881
0.84	4,5-Dihydroorotic acid	1.00 ± 0.11	0.93 ± 0.01	0.90 ± 0.06	0.2766	0.5304	0.2577	0.8073
0.84	5-Methylcytidine	0.99 ± 0.02	1.00 ± 0.01	1.00 ± 0.01	0.9073	0.9621	0.8997	0.9831
0.84	Glucose 6-phosphate	0.93 ± 0.08	0.96 ± 0.13	0.90 ± 0.13	0.8530	0.9586	0.9539	0.8397
0.84	Glutamic acid	0.98 ± 0.13	0.97 ± 0.06	0.97 ± 0.05	0.9925	0.9923	0.9959	0.9994
0.84	Mannitol	1.03 ± 0.08	1.12 ± 0.12	1.16 ± 0.07	0.3100	0.5239	0.2969	0.8710
0.85	Creatinine	1.05 ± 0.05	0.94 ± 0.07	1.01 ± 0.08	0.2006	0.1878	0.7938	0.4163
0.85	Orotic acid	1.04 ± 0.06	0.96 ± 0.11	0.94 ± 0.06	0.3392	0.4915	0.3430	0.9446
0.85	Ribose	1.06 ± 0.04	1.06 ± 0.03	0.97 ± 0.07	0.1014	1.0000	0.1368	0.1372

0.87	Glycolic acid	1.02 ± 0.08	1.06 ± 0.16	1.09 ± 0.10	0.7610	0.9176	0.7414	0.9326
0.89	3-Methylindole	1.07 ± 0.11	0.93 ± 0.11	0.92 ± 0.11	0.2676	0.3521	0.3027	0.9910
0.89	Creatine	1.07 ± 0.11	0.93 ± 0.11	0.92 ± 0.11	0.2608	0.3454	0.2952	0.9904
0.89	Ribose 5-phosphate	0.90 ± 0.12	1.00 ± 0.17	0.93 ± 0.25	0.7993	0.7960	0.9849	0.8788
0.90	Glycerol 3-phosphate	0.95 ± 0.07	1.05 ± 0.18	0.98 ± 0.18	0.7260	0.7208	0.9766	0.8330
0.91	N-Acetyllactosamine	0.83 ± 0.15	0.86 ± 0.12	0.84 ± 0.21	0.9789	0.9771	0.9963	0.9917
0.91	Trimethylamine N-oxide	1.02 ± 0.03	1.04 ± 0.08	1.06 ± 0.07	0.7951	0.9556	0.7792	0.9170
0.95	Deoxycytidine	1.02 ± 0.21	0.91 ± 0.19	0.94 ± 0.08	0.7425	0.7340	0.8525	0.9729
0.96	Threitol	0.87 ± 0.07	0.74 ± 0.13	0.89 ± 0.06	0.1873	0.2775	0.9710	0.2084
0.98	Valine	1.01 ± 0.01	0.98 ± 0.02	0.99 ± 0.01	0.0771	0.0694	0.2419	0.6177
1.04	3-Aminoisobutanoate	0.91 ± 0.07	1.02 ± 0.12	1.03 ± 0.12	0.3670	0.4644	0.3986	0.9894
1.04	3-Pyridylacetic acid	0.85 ± 0.04	1.03 ± 0.06	1.06 ± 0.15	0.0577	0.1088	0.0655	0.9166
1.04	UDP-N-acetylglucosamine	1.00 ± 0.01	1.05 ± 0.04	1.05 ± 0.07	0.3322	0.4269	0.3648	0.9894
1.20	Malic acid	1.06 ± 0.03	1.11 ± 0.07	1.05 ± 0.02	0.2496	0.4035	0.9164	0.2508
1.20	Pyruvic acid	1.03 ± 0.02	1.10 ± 0.06	1.06 ± 0.03	0.1768	0.1603	0.7045	0.4336

1.36	Acetylcarnitine	0.83 ± 0.09	0.78 ± 0.02	0.92 ± 0.04	0.0581	0.6089	0.1855	0.0530
1.38	Lactate	1.03 ± 0.06	0.97 ± 0.05	1.01 ± 0.05	0.4008	0.3796	0.8659	0.6454
1.39	1-Methyladenine	0.96 ± 0.06	1.01 ± 0.07	1.07 ± 0.12	0.4224	0.7701	0.3928	0.7674
1.39	Methionine	0.93 ± 0.02	0.98 ± 0.06	0.96 ± 0.01	0.2426	0.2213	0.5321	0.7389
1.50	2-Oxoglutarate	1.05 ± 0.02	1.12 ± 0.09	1.17 ± 0.12	0.2953	0.6201	0.2698	0.7391
1.50	Citric acid	1.05 ± 0.02	1.12 ± 0.09	1.17 ± 0.12	0.2955	0.6177	0.2701	0.7419
1.50	Norepinephrine	0.96 ± 0.08	0.96 ± 0.06	0.95 ± 0.06	0.9406	0.9965	0.9646	0.9400
1.54	Pyroglutamic acid	0.74 ± 0.23	0.68 ± 0.21	0.74 ± 0.28	0.9489	0.9560	0.9999	0.9591
1.55	Pyrrolidonecarboxylic acid	0.81 ± 0.23	0.74 ± 0.18	0.79 ± 0.24	0.9276	0.9243	0.9927	0.9622
1.83	Fumarate	1.06 ± 0.02	0.99 ± 0.03	1.03 ± 0.13	0.5733	0.5462	0.8758	0.8213
1.92	Norspermidine	0.89 ± 0.01	0.93 ± 0.03	0.97 ± 0.05	0.0680	0.2763	0.0589	0.4830
1.93	Isoleucine	1.00 ± 0.01	1.00 ± 0.01	1.00 ± 0.01	0.6714	0.8602	0.6489	0.9206
1.96	Tyrosine	0.94 ± 0.03	1.06 ± 0.05	1.11 ± 0.07	0.0195*	0.0645	0.0188*	0.5847
2.16	Norleucine	1.00 ± 0.01	0.99 ± 0.01	0.99 ± 0.02	0.7860	0.9347	0.7679	0.9333
2.67	Propionylcarnitine	0.84 ± 0.02	0.94 ± 0.12	1.02 ± 0.08	0.1048	0.4284	0.0907	0.4662

3.48	Phenylethanolamine	1.25 ± 0.24	1.21 ± 0.14	1.26 ± 0.27	0.9501	0.9615	0.9998	0.9557
3.80	Phenylalanine	1.01 ± 0.01	1.01 ± 0.01	1.01 ± 0.01	0.9952	0.9947	0.9987	0.9986
4.17	Thymidine	1.03 ± 0.08	1.11 ± 0.13	1.15 ± 0.04	0.2906	0.5379	0.2722	0.8233
4.73	Tryptophan	0.95 ± 0.02	1.00 ± 0.02	1.02 ± 0.03	0.0294*	0.1058	0.0270*	0.5429
4.74	1-Phenylethylamine	0.91 ± 0.04	1.00 ± 0.01	1.11 ± 0.10	0.0190*	0.2401	0.0158*	0.1448
4.74	3-Methylglutaric acid	0.91 ± 0.03	0.98 ± 0.05	1.11 ± 0.07	0.0105*	0.3559	0.0094**	0.0518
4.84	γ-Glutamylleucine	0.93 ± 0.07	1.08 ± 0.04	1.07 ± 0.06	0.0360*	0.0447*	0.0645	0.9530
4.85	4-Hydroxybenzaldehyde	1.03 ± 0.06	1.03 ± 0.06	1.11 ± 0.05	0.2285	0.9951	0.3004	0.2678
4.85	Homovanillate	1.03 ± 0.06	1.03 ± 0.06	1.11 ± 0.06	0.2451	0.9952	0.3192	0.2852
5.34	Valerylcarnitine	0.86 ± 0.08	0.85 ± 0.11	1.00 ± 0.05	0.1388	0.9921	0.1967	0.1690
5.43	Ketoleucine	1.03 ± 0.10	0.95 ± 0.08	1.04 ± 0.05	0.3799	0.5057	0.9719	0.3951
6.93	Indolelactic acid	1.04 ± 0.04	1.08 ± 0.08	1.08 ± 0.05	0.6910	0.7397	0.7297	0.9998
12.35	Hexadecanoylcarnitine	0.99 ± 0.03	1.13 ± 0.07	1.07 ± 0.09	0.1090	0.0950	0.3889	0.5299

[a] ABR, Abraxane®; CTL, control; PHY, Phyxol; PTX, paclitaxel; RT, retention time; *, $p < 0.05$; **, $p < 0.01$.

[b] Since isomers could not be distinguished by the present analytical method, the metabolites listed herein were representative.

[c] Three independent samples were prepared for each condition in each experiment. Each sample was analyzed twice by ultra-high performance liquid chromatography-quadrupole time-of-flight mass spectrometry. Three independent experiments were conducted. Data are expressed as the fold change relative to the control group and are presented as mean ± standard deviation (n = 3).

[d] One-way analysis of variance was used to analyze the difference in fold changes; all pairs, Tukey HSD (honestly significant difference) post-hoc test comparisons were then performed.
