# Supplementary material for: Cell metabolomics analyses revealed a role of altered fatty acid oxidation in neurotoxicity pattern difference between nab-paclitaxel and solvent-based paclitaxel
Source: PLoS One. 2021 Mar 19;16(3):e0248942. doi: 10.1371/journal.pone.0248942 (PMC7978375; doi:10.1371/journal.pone.0248942)
Supplement: S1 File — (PDF) [file pone.0248942.s003.pdf]

Original images for Fig 4.

The representative Western blot image shown in Fig 4A (results for three of the six independent samples for each condition)

Medium-chain acyl-CoA dehydrogenase (MCAD)

From left to right: Control, Control, Control, Paclitaxel, Paclitaxel, Paclitaxel, Phyxol, Phyxol, Phyxol, Abraxane®, Abraxane®, Abraxane®

Cyclophilin A

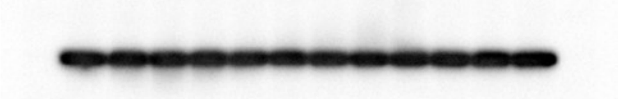

From left to right: Control, Control, Control, Paclitaxel, Paclitaxel, Paclitaxel, Phyxol, Phyxol, Phyxol, Abraxane®, Abraxane®, Abraxane®

The other Western blot image (results for three of the six independent samples for each condition)

Medium-chain acyl-CoA dehydrogenase (MCAD)

From left to right: Control, Control, Control, Paclitaxel, Paclitaxel, Paclitaxel, Phyxol,

Phyxol, Phyxol, Abraxane®, Abraxane®, Abraxane®

Cyclophilin A

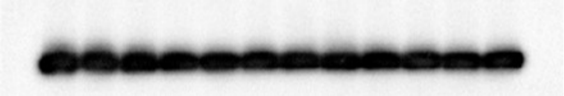

From left to right: Control, Control, Control, Paclitaxel, Paclitaxel, Paclitaxel, Phyxol, Phyxol, Phyxol, Abraxane®, Abraxane®, Abraxane®

The representative Western blot image shown in Fig 4B (results for three of the six independent samples for each condition)

Medium-chain acyl-CoA dehydrogenase (MCAD)

From left to right: Control, Control, Control, Paclitaxel, Paclitaxel, Paclitaxel, Phyxol, Phyxol, Phyxol, Abraxane®, Abraxane®, Abraxane®

Cyclophilin A

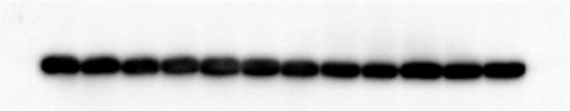

From left to right: Control, Control, Control, Paclitaxel, Paclitaxel, Paclitaxel, Phyxol, Phyxol, Phyxol, Abraxane®, Abraxane®, Abraxane®

The other Western blot image (results for three of the six independent samples for each condition)

Medium-chain acyl-CoA dehydrogenase (MCAD)

From left to right: Control, Control, Control, Paclitaxel, Paclitaxel, Paclitaxel, Phyxol, Phyxol, Phyxol, Abraxane®, Abraxane®, Abraxane®

Cyclophilin A

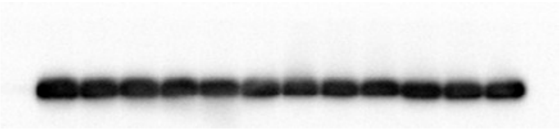

From left to right: Control, Control, Control, Paclitaxel, Paclitaxel, Paclitaxel, Phyxol, Phyxol, Phyxol, Abraxane®, Abraxane®, Abraxane®
