# Supplementary material for: Cell metabolomics analyses revealed a role of altered fatty acid oxidation in neurotoxicity pattern difference between nab-paclitaxel and solvent-based paclitaxel
Source: PLoS One. 2021 Mar 19;16(3):e0248942. doi: 10.1371/journal.pone.0248942 (PMC7978375; doi:10.1371/journal.pone.0248942)
Supplement: S2 File — Data for figures. (PDF) [file pone.0248942.s004.pdf]

Details regarding experimental procedures. Data for figures.

Materials and methods

Additional information for SH-SY5Y cells

Passage numbers: 10-15

The date for obtaining the cells from Prof. Ming-Fu Chang's laboratory: August 22, 2018

Concentration normalization for mass spectrometry analyses of metabolic profiles

In order to conduct concentration normalization, an Agilent 1290 UHPLC system (ultra-high performance liquid chromatography) (Agilent Technologies, Santa Clara, CA, USA) coupled to an Agilent 6460 QqQ (triple quadrupole) mass spectrometry (Agilent Technologies, Santa Clara, CA, USA) was utilized. The procedure was similar to that previously described by Chen et al. [1] with necessary adjustments. A sample of 2 μL was injected into electrospray ionization mass spectrometry to conduct flow injection analysis. The ion suppression indicator (ISI), hexakis(2,2,3,3-tetrafluoropropoxy)phosphazene, was spiked in the blank solution and the cell extracts. The mobile phase consisted of a 1:1 mixture of water/0.1% formic acid and acetonitrile/0.1% formic acid. The flow rate was 200 μL min^{-1}. For sample ionization, a Jet Stream electrospray ionization source was applied. The mass spectrometer

parameters were as follows: 100°C gas temperature, 3 L min^{-1} gas flow, 10 psi nebulizer pressure, 150°C sheath gas temperature, 3 L min^{-1} sheath gas flow, 4 kV capillary voltage in the positive ion mode, and 120 V fragmentation voltage. The calibration curve was established by signal reduction (the extent of ISI in blank solution subtracted by that in cell samples) and dilution factors of the cell samples.

Concentration normalization for mass spectrometry analyses of long-chain and very long-chain fatty acids

For concentration normalization, an Agilent 1290 UHPLC system coupled to an Agilent 6460 QqQ mass spectrometry was utilized. The procedure was similar to that previously described by Chao et al. [2] with necessary adjustments. A sample of 2 μL was injected into electrospray ionization mass spectrometry to conduct flow injection analysis. The mobile phase consisted of a mixture of solvent A and solvent B (1:1). The former contained 40% acetonitrile, 0.1% formic acid, 10 mM ammonium acetate while the latter was a mixture of isopropanol:acetonitrile (9:1), 0.1% formic acid, 10 mM ammonium acetate. The flow rate was 200 μL min^{-1}. A Jet Stream electrospray ionization source was applied for sample ionization. The mass spectrometer parameters were as follows: 350°C gas temperature, 10 L min^{-1} gas flow, 40 psi nebulizer pressure, 350°C sheath gas temperature, 11 L min^{-1} sheath gas flow, 4 kV

capillary voltage in the positive ion mode, and 120 V fragmentation voltage. The calibration curve was established by the dilution factors of the cell samples and the reciprocal of the total ion chromatogram area in the precursor ion scan of m/z 184.

References

1. Chen GY, Liao HW, Tsai IL, Tseng YJ, Kuo CH. Using the matrix-induced ion suppression method for concentration normalization in cellular metabolomics studies. Anal Chem. 2015;87:9731-9739.

2. Chao HC, Chen GY, Hsu LC, Liao HW, Yang SY, Wang SY, et al. Using precursor ion scan of 184 with liquid chromatography-electrospray ionization-tandem mass spectrometry for concentration normalization in cellular lipidomic studies. Anal Chim Acta. 2017;971:68-77.

Data for Fig 1

(A)

Cell viability (% of control cell growth) of undifferentiated SH-SY5Y cells treated with 100 nM of paclitaxel, Phyxol, or Abraxane® for 24 hours assessed by the sulforhodamine B (SRB) assay.

Paclitaxel (Experiments 1-3)	Phyxol (Experiments 1-3)	Abraxane® (Experiments 1-3)
87.3	79.6	87.7
75.4	75.1	69.3
76.5	71.9	83.3

$F(2,6) = 0.3881$; $p = 0.6942$

(B)

Cell viability (% of control cell growth) of differentiated SH-SY5Y cells treated with 100 nM of paclitaxel, Phyxol, or Abraxane® for 24 hours assessed by the sulforhodamine B (SRB) assay.

Paclitaxel (Experiments 1-3)	Phyxol (Experiments 1-3)	Abraxane® (Experiments 1-3)
96.5	101.6	97.5
100.2	102.0	102.4
98.9	96.0	100.0

$F(2,6) = 0.2668$; $p = 0.7744$

Data for Fig 2

(A)

Fold changes relative to the control group in the levels of carnitine and acylcarnitines of undifferentiated SH-SY5Y cells treated with 100 nM of paclitaxel (in dimethyl sulfoxide), Phyxol, or Abraxane® for 24 hours.

		Paclitaxel	
	Experiment 1	Experiment 2	Experiment 3
Carnitine	0.571	0.684	0.632
Acetylcarnitine	0.609	0.518	0.648
Propionylcarnitine	0.781	0.783	0.741
Iso-/Butyrylcarnitine	0.648	0.758	0.720
Isovaleryl-/2-Methylbutyryl-/Valerylcarnitine	0.586	0.603	0.661
Tiglylcarnitine	0.567	0.658	0.681
Hexanoylcarnitine	0.682	0.685	0.633
Octanoylcarnitine	0.677	0.809	0.968
Decanoylcarnitine	0.808	0.822	0.841
Tetradecanoylcarnitine	0.625	0.779	0.750
Tetradecenoylcarnitine	0.616	0.687	0.821
Hexadecanoylcarnitine	0.715	0.849	0.981
Hexadecenoylcarnitine	0.710	0.843	0.875
Stearoylcarnitine	0.545	0.812	0.870
Oleoylcarnitine	0.804	1.125	0.968
		Phyxol	
	Experiment 1	Experiment 2	Experiment 3
Carnitine	0.567	0.461	0.604
Acetylcarnitine	0.607	0.645	0.688
Propionylcarnitine	0.556	0.708	0.893
Iso-/Butyrylcarnitine	0.550	0.659	0.751

Isovaleryl-/2-Methylbutyryl-/Valerylcarnitine	0.580	0.611	0.752
Tiglylcarnitine	0.516	0.600	0.881
Hexanoylcarnitine	0.462	0.544	0.651
Octanoylcarnitine	3.160	2.365	2.807
Decanoylcarnitine	0.663	0.758	0.476
Tetradecanoylcarnitine	0.627	0.665	0.837
Tetradecenoylcarnitine	0.638	0.714	0.851
Hexadecanoylcarnitine	0.853	0.969	0.959
Hexadecenoylcarnitine	0.830	0.853	0.999
Stearoylcarnitine	0.834	0.803	0.908
Oleoylcarnitine	1.022	1.055	1.051
		Abraxane®	
	Experiment 1	Experiment 2	Experiment 3
Carnitine	0.835	0.794	0.797
Acetylcarnitine	0.892	0.832	0.811
Propionylcarnitine	1.065	1.056	1.064
Iso-/Butyrylcarnitine	0.881	0.852	0.909
Isovaleryl-/2-Methylbutyryl-/Valerylcarnitine	0.887	0.801	0.951
Tiglylcarnitine	0.842	0.827	0.954
Hexanoylcarnitine	0.978	0.867	1.025
Octanoylcarnitine	1.055	1.142	1.114
Decanoylcarnitine	0.976	1.040	1.275
Tetradecanoylcarnitine	0.923	0.930	0.932
Tetradecenoylcarnitine	0.915	0.964	1.096
Hexadecanoylcarnitine	1.174	1.166	1.106
Hexadecenoylcarnitine	1.101	1.037	1.067
Stearoylcarnitine	1.009	1.059	1.127
Oleoylcarnitine	1.324	1.294	1.252

Carnitine

$F(2,6) = 17.7333$; $p = 0.0030$

		p value
Paclitaxel	Phyxol	0.2264
Paclitaxel	Abraxane®	0.0175
Phyxol	Abraxane®	0.0027

Acetylcarnitine

F(2,6) = 20.3823; *p* = 0.0021

		p value
Paclitaxel	Phyxol	0.4355
Paclitaxel	Abraxane®	0.0022
Phyxol	Abraxane®	0.0075

Propionylcarnitine

F(2,6) = 10.5978; *p* = 0.0107

		p value
Paclitaxel	Phyxol	0.8208
Paclitaxel	Abraxane®	0.0250
Phyxol	Abraxane®	0.0126

Iso-/Butyrylcarnitine

F(2,6) = 9.0577; *p* = 0.0154

		p value
Paclitaxel	Phyxol	0.6095
Paclitaxel	Abraxane®	0.0487
Phyxol	Abraxane®	0.0153

Isovaleryl-/2-Methylbutyryl-/Valerylcarnitine

$F(2,6) = 11.8736$; $p = 0.0082$

		p value
Paclitaxel	Phyxol	0.8605
Paclitaxel	Abraxane®	0.0102
Phyxol	Abraxane®	0.0181

Tiglylcarnitine

$F(2,6) = 3.3891$; $p = 0.1035$

Hexanoylcarnitine

$F(2,6) = 23.9058$; $p = 0.0014$

		p value
Paclitaxel	Phyxol	0.2204
Paclitaxel	Abraxane®	0.0071
Phyxol	Abraxane®	0.0013

Octanoylcarnitine

$F(2,6) = 55.4092$; $p = 0.0001$

		p value
Paclitaxel	Phyxol	0.0002
Paclitaxel	Abraxane®	0.3899
Phyxol	Abraxane®	0.0004

Decanoylcarnitine

$F(2,6) = 10.7925$; $p = 0.0103$

		p value
Paclitaxel	Phyxol	0.2182
Paclitaxel	Abraxane®	0.0772
Phyxol	Abraxane®	0.0086

Tetradecanoylcarnitine

$F(2,6) = 7.1601$; $p = 0.0257$

		p value
Paclitaxel	Phyxol	0.9919
Paclitaxel	Abraxane®	0.0418
Phyxol	Abraxane®	0.0361

Tetradecenoylcarnitine

$F(2,6) = 7.0395$; $p = 0.0267$

		p value
Paclitaxel	Phyxol	0.9479
Paclitaxel	Abraxane®	0.0337
Phyxol	Abraxane®	0.0492

Hexadecanoylcarnitine

$F(2,6) = 9.4056$; $p = 0.0141$

		p value
Paclitaxel	Phyxol	0.5503
Paclitaxel	Abraxane®	0.0137
Phyxol	Abraxane®	0.0489

Hexadecenoylcarnitine

F(2,6) = 9.2002; *p* = 0.0149

		p value
Paclitaxel	Phyxol	0.4081
Paclitaxel	Abraxane®	0.0133
Phyxol	Abraxane®	0.0674

Stearoylcarnitine

F(2,6) = 6.6749; *p* = 0.0298

		p value
Paclitaxel	Phyxol	0.5070
Paclitaxel	Abraxane®	0.0269
Phyxol	Abraxane®	0.1150

Oleoylcarnitine

F(2,6) = 9.4090; *p* = 0.0141

		p value
Paclitaxel	Phyxol	0.6149
Paclitaxel	Abraxane®	0.0142
Phyxol	Abraxane®	0.0441

(B)

Fold changes relative to the control group in the levels of carnitine and acylcarnitines of differentiated SH-SY5Y cells treated with 100 nM of paclitaxel (in dimethyl sulfoxide), Phyxol, or Abraxane® for 24 hours.

		Paclitaxel	
	Experiment 1	Experiment 2	Experiment 3
Carnitine	0.865	0.912	0.954
Acetylcarnitine	0.707	0.705	0.787
Propionylcarnitine	0.917	1.025	0.869
Iso-/Butyrylcarnitine	0.912	0.945	0.914
Isovaleryl-/2-Methylbutyryl-/Valerylcarnitine	0.817	0.880	0.829
Tiglylcarnitine	0.856	0.966	0.968
Hexanoylcarnitine	0.917	0.860	0.919
Octanoylcarnitine	0.888	0.763	1.007
Decanoylcarnitine	0.912	1.068	0.884
Dodecanoylcarnitine	1.092	1.137	0.890
Tetradecanoylcarnitine	0.829	1.037	0.905
Tetradecenoylcarnitine	0.992	1.012	0.864
Hexadecanoylcarnitine	0.722	0.894	0.820
Hexadecenoylcarnitine	0.829	0.958	0.854
Stearoylcarnitine	0.529	0.529	0.530
Oleoylcarnitine	0.743	0.839	0.742
		Phyxol	
	Experiment 1	Experiment 2	Experiment 3
Carnitine	0.994	1.198	0.993
Acetylcarnitine	0.793	0.843	0.992
Propionylcarnitine	1.120	1.001	0.888
Iso-/Butyrylcarnitine	0.943	1.018	0.873
Isovaleryl-/2-Methylbutyryl-/Valerylcarnitine	0.926	1.024	0.846
Tiglylcarnitine	0.916	1.070	0.973
Hexanoylcarnitine	1.038	1.050	1.043
Octanoylcarnitine	2.933	2.298	3.333
Decanoylcarnitine	0.769	1.025	1.036
Dodecanoylcarnitine	0.906	1.245	0.932
Tetradecanoylcarnitine	0.963	1.099	0.907
Tetradecenoylcarnitine	1.011	1.110	0.926
Hexadecanoylcarnitine	0.953	1.032	0.923

Hexadecenoylcarnitine	0.959	1.080	0.954
Stearoylcarnitine	0.769	0.715	0.747
Oleoylcarnitine	0.947	1.050	0.873
		Abraxane®	
	Experiment 1	Experiment 2	Experiment 3
Carnitine	1.095	1.087	1.104
Acetylcarnitine	1.007	0.874	1.119
Propionylcarnitine	1.237	1.122	1.134
Iso-/Butyrylcarnitine	1.085	1.095	1.093
Isovaleryl-/2-Methylbutyryl-/Valerylcarnitine	1.061	1.109	1.080
Tiglylcarnitine	1.100	1.111	1.105
Hexanoylcarnitine	1.100	0.968	1.054
Octanoylcarnitine	1.203	0.841	1.245
Decanoylcarnitine	0.870	1.011	1.266
Dodecanoylcarnitine	0.976	1.292	1.192
Tetradecanoylcarnitine	1.034	1.163	1.106
Tetradecenoylcarnitine	1.044	1.131	1.109
Hexadecanoylcarnitine	0.882	0.968	1.013
Hexadecenoylcarnitine	0.963	1.117	1.070
Stearoylcarnitine	0.667	0.592	0.760
Oleoylcarnitine	0.864	0.975	0.958

Carnitine

F(2,6) = 5.4565; $p = 0.0446$

		p value
Paclitaxel	Phyxol	0.0970
Paclitaxel	Abraxane®	0.0481
Phyxol	Abraxane®	0.8458

Acetylcarnitine

F(2,6) = 5.7663; p = 0.0401

		p value
Paclitaxel	Phyxol	0.2442
Paclitaxel	Abraxane®	0.0337
Phyxol	Abraxane®	0.3230

Propionylcarnitine

F(2,6) = 5.1327; p = 0.0502

Iso-/Butyrylcarnitine

F(2,6) = 13.2659; p = 0.0063

		p value
Paclitaxel	Phyxol	0.8262
Paclitaxel	Abraxane®	0.0077
Phyxol	Abraxane®	0.0145

Isovaleryl-/2-Methylbutyryl-/Valerylcarnitine

F(2,6) = 13.9254; p = 0.0056

		p value
Paclitaxel	Phyxol	0.2069
Paclitaxel	Abraxane®	0.0047
Phyxol	Abraxane®	0.0388

Tiglylcarnitine

F(2,6) = 7.0988; p = 0.0262

		p value
Paclitaxel	Phyxol	0.5060
Paclitaxel	Abraxane®	0.0238
Phyxol	Abraxane®	0.1009

Hexanoylcarnitine

F(2,6) = 10.9949; p = 0.0099

		p value
Paclitaxel	Phyxol	0.0150
Paclitaxel	Abraxane®	0.0163
Phyxol	Abraxane®	0.9966

Octanoylcarnitine

F(2,6) = 31.2535; p = 0.0007

		p value
Paclitaxel	Phyxol	0.0009
Paclitaxel	Abraxane®	0.7336
Phyxol	Abraxane®	0.0016

Decanoylcarnitine

F(2,6) = 0.4174; p = 0.6765

Dodecanoylcarnitine

F(2,6) = 0.5466; p = 0.6053

Tetradecanoylcarnitine

F(2,6) = 2.8877; p = 0.1323

Tetradecenoylcarnitine

F(2,6) = 2.5616; p = 0.1570

Hexadecanoylcarnitine

F(2,6) = 4.4704; p = 0.0648

Hexadecenoylcarnitine

F(2,6) = 4.2315; p = 0.0714

Stearoylcarnitine

F(2,6) = 13.7541; p = 0.0057

		p value
Paclitaxel	Phyxol	0.0051
Paclitaxel	Abraxane®	0.0315
Phyxol	Abraxane®	0.2818

Oleoylcarnitine

F(2,6) = 5.9958; p = 0.0371

		p value
Paclitaxel	Phyxol	0.0432
Paclitaxel	Abraxane®	0.0730
Phyxol	Abraxane®	0.9070

Data for Fig 3

(A)

Fold changes relative to the control group in the levels of long-chain and very long-chain fatty acids of undifferentiated SH-SY5Y cells treated with 100 nM of paclitaxel (in dimethyl sulfoxide), Phyxol, or Abraxane® for 24 hours.

		Paclitaxel	
	Experiment 1	Experiment 2	Experiment 3
Myristic acid	0.777	0.949	0.930
Palmitic acid	0.916	1.066	0.893
Palmitoleic acid	0.895	0.911	0.839
Stearic acid	0.881	1.059	0.952
Oleic acid	0.748	0.899	0.830
Linoleic acid	0.713	0.898	0.794
Eicosanoic acid	0.896	1.073	0.983
		Phyxol	
	Experiment 1	Experiment 2	Experiment 3
Myristic acid	0.825	0.851	0.903
Palmitic acid	1.103	1.059	1.121
Palmitoleic acid	0.980	1.018	1.029
Stearic acid	1.117	1.101	1.199
Oleic acid	0.876	0.915	1.048
Linoleic acid	0.997	0.993	1.026
Eicosanoic acid	0.994	1.105	1.020
		Abraxane®	

	Experiment 1	Experiment 2	Experiment 3
Myristic acid	1.064	1.076	1.067
Palmitic acid	1.346	1.212	1.228
Palmitoleic acid	1.141	1.171	1.258
Stearic acid	1.206	1.081	1.187
Oleic acid	0.987	0.930	1.005
Linoleic acid	1.352	1.136	1.241
Eicosanoic acid	1.164	1.238	1.262

Myristic acid

$F(2,6) = 11.1640$; $p = 0.0095$

		p value
Paclitaxel	Phyxol	0.8573
Paclitaxel	Abraxane®	0.0210
Phyxol	Abraxane®	0.0116

Palmitic acid

$F(2,6) = 13.6140$; $p = 0.0059$

		p value
Paclitaxel	Phyxol	0.1260
Paclitaxel	Abraxane®	0.0048
Phyxol	Abraxane®	0.0636

Palmitoleic acid

$F(2,6) = 37.5968$; $p = 0.0004$

		p value
Paclitaxel	Phyxol	0.0277
Paclitaxel	Abraxane®	0.0003

Phyxol	Abraxane®	0.0055

Stearic acid

F(2,6) = 6.7109; p = 0.0295

		p value
Paclitaxel	Phyxol	0.0544
Paclitaxel	Abraxane®	0.0368
Phyxol	Abraxane®	0.9454

Oleic acid

F(2,6) = 3.6412; p = 0.0922

Linoleic acid

F(2,6) = 21.3451; p = 0.0019

		p value
Paclitaxel	Phyxol	0.0537
Paclitaxel	Abraxane®	0.0015
Phyxol	Abraxane®	0.0291

Eicosanoic acid

F(2,6) = 9.9750; p = 0.0124

		p value
Paclitaxel	Phyxol	0.6035
Paclitaxel	Abraxane®	0.0125
Phyxol	Abraxane®	0.0391

(B)

Fold changes relative to the control group in the levels of long-chain and very long-chain fatty acids of differentiated SH-SY5Y cells treated with 100 nM of paclitaxel (in dimethyl sulfoxide), Phyxol, or Abraxane® for 24 hours.

		Paclitaxel	
	Experiment 1	Experiment 2	Experiment 3
Palmitic acid	1.121	1.132	1.264
Stearic acid	1.104	0.952	1.114
Oleic acid	1.176	1.165	1.272
Linoleic acid	0.982	0.992	1.156
Eicosanoic acid	1.048	1.100	1.223
Tetracosanoic acid	1.089	1.095	1.236
Hexacosanoic acid	1.171	1.252	1.291
		Phyxol	
	Experiment 1	Experiment 2	Experiment 3
Palmitic acid	1.074	1.187	1.220
Stearic acid	1.158	1.002	1.141
Oleic acid	1.083	1.043	1.217
Linoleic acid	0.930	1.004	1.071
Eicosanoic acid	1.146	1.085	1.030
Tetracosanoic acid	0.940	0.996	1.033
Hexacosanoic acid	0.995	1.141	1.161
		Abraxane®	
	Experiment 1	Experiment 2	Experiment 3
Palmitic acid	1.274	1.294	1.180
Stearic acid	1.148	1.047	1.005
Oleic acid	1.228	1.128	1.088
Linoleic acid	1.012	1.041	0.908
Eicosanoic acid	1.173	1.255	1.107

Tetracosanoic acid	1.199	1.112	1.129
Hexacosanoic acid	1.132	1.176	1.059

Palmitic acid

F(2,6) = 1.3149; p = 0.3361

Stearic acid

F(2,6) = 0.2238; p = 0.8059

Oleic acid

F(2,6) = 1.0891; p = 0.3949

Linoleic acid

F(2,6) = 0.3994; p = 0.6873

Eicosanoic acid

F(2,6) = 1.1224; p = 0.3854

Tetracosanoic acid

F(2,6) = 6.3065; p = 0.0335

		p value
Paclitaxel	Phyxol	0.0541
Paclitaxel	Abraxane®	0.9895
Phyxol	Abraxane®	0.0456

Hexacosanoic acid

$F(2,6) = 3.2233$; $p = 0.1120$

Data for Fig 4

(A)

Fold changes in the medium-chain acyl-CoA dehydrogenase (MCAD) protein level relative to control (relative expression normalized to cyclophilin A) in undifferentiated SH-SY5Y cells treated with 100 nM of paclitaxel (in dimethyl sulfoxide), Phyxol, or Abraxane® for 24 hours

Paclitaxel (Samples 1-6)	Phyxol (Samples 1-6)	Abraxane® (Samples 1-6)
1.144	0.941	1.066
1.049	0.870	1.130
0.924	0.918	0.979
0.977	0.915	1.120
1.052	0.811	0.921
1.033	0.823	0.982

$F(2,15) = 8.8081$; $p = 0.0030$

		p value
Paclitaxel	Phyxol	0.0070
Paclitaxel	Abraxane®	0.9972

Phyxol	Abraxane®	0.0061

(B)

Fold changes in the medium-chain acyl-CoA dehydrogenase (MCAD) protein level relative to control (relative expression normalized to cyclophilin A) in differentiated SH-SY5Y cells treated with 100 nM of paclitaxel (in dimethyl sulfoxide), Phyxol, or Abraxane® for 24 hours

Paclitaxel (Samples 1-6)	Phyxol (Samples 1-6)	Abraxane® (Samples 1-6)
1.005	0.883	1.056
1.006	0.921	1.015
1.077	0.963	0.990
1.073	0.833	1.069
1.055	0.924	1.029
1.030	0.793	1.065

$F(2,15) = 23.2812$; $p = 0.00003$

		p value
Paclitaxel	Phyxol	0.0001
Paclitaxel	Abraxane®	0.9889
Phyxol	Abraxane®	0.0001
